# Emergence of High Level Carbapenem and Extensively Drug Resistant *Escherichia coli* ST746 Producing NDM-5 in Influent of Wastewater Treatment Plant, Seoul, South Korea

**DOI:** 10.3389/fmicb.2021.645411

**Published:** 2021-03-23

**Authors:** Hanseob Shin, Yeonghyeon Kim, Dukki Han, Hor-Gil Hur

**Affiliations:** ^1^School of Earth Sciences and Environmental Engineering, Gwangju Institute of Science and Technology, Gwangju, South Korea; ^2^Department of Marine Molecular Bioscience, Gangneung-Wonju National University, Gangneung, South Korea

**Keywords:** carbapenem resistance, extensively drug resistance, blaNDM gene, wastewater treatment plants, *Escherichia coli*, horizontal gene transfer, aquatic environment

## Abstract

High level carbapenem and extensively drug resistant (XDR) *Escherichia coli* strain N7, which produces a variant of New Delhi metallo-β-lactamase (NDM-5), was isolated from the influent of the Jungnang wastewater treatment plant located on Han River, Seoul, South Korea. Phenotypic and genotypic resistances to carbapenem were tested using agar and broth dilution methods, and polymerase chain reaction. Whole-genome sequencing was performed to characterize the genetic structure of strain N7. *E. coli* strain N7, which harbors the *bla*_*NDM–*5_ gene, showed high level of carbapenem resistance at concentrations of doripenem (512 mg/L) and meropenem (256 mg/L), and XDR to 15 antibiotics. Based on the genomic sequence analysis, two plasmids, a hybrid IncHI2/N-type and an IncX3 type, were present. The former contains a cluster (*bla*_*NDM–*5_-*ble*_*MBL*_-*trpF*-*dsbD*) bracketed by multi-insertional sequences, IS*3000*, IS*Aba125*, IS*5*, and IS*26*. The latter carries the following resistance genes: *bla*_*CTX–*14_, *aac(3)-IV, aadA1, aadA2, aph(3′)-Ia, aph(4)-Ia, sul1, sul2, sul3, dfrA12, fosA3, oqxA, oqxB, mph(A)*, and *floR*, and *cmlA1*. The chromosome, contig3, and contig5 also carry *bla*_*CTX–*64_ and *mdf(A), tet(A)*, and *erm(B), tet(M)* and *aadA22*, respectively. Strain N7 also harbors virulence factors such as *fimH*, *flu*, *ecpABCDE*, *sfmA, hlyE*, and *gadA*. This study demonstrates the emergence of high level carbapenem resistant XDR *E. coli* strain N7 containing *bla*_*NDM–*5_ in aquatic environment, Seoul, South Korea. Due to the presence of mobile genetic elements, this strain could horizontally transfer resistance genes, including *bla*_*NDM–*5_ to environmental bacteria. Thus, it is necessary to conduct continuous surveillance for carbapenem resistance in various aquatic environments.

## Introduction

Carbapenem-resistant *Enterobacteriaceae* (CRE) is one of the most critical pathogens, together with carbapenem-resistant *Acinetobacter baumannii* and *Pseudomonas aeruginosa*, and has been clinically issued with growing concerns in need of new antibiotics (Tacconelli et al., 2017). CRE can produce several enzymes belonging to the class of New Delhi metallo-β-lactamase (NDM) to hydrolyze carbapenems (Doi and Paterson, 2015). Since the first report of NDM (Yong et al., 2009), a series of NDM variants, which possess distinct hydrolytic activity against β-lactams (*bla*_*NDM*_) from NDM-1 to NDM-29, have been identified with the clinical evolution of NDM (Cheng et al., 2018). In particular, NDM-5 producing *Escherichia coli* shows higher level of resistance to carbapenems compared to previously reported NDM-1 producing bacteria (Hornsey et al., 2011).

The first occurrence of NDM-5 producing *E. coli* EC405 was reported in a patient in the United Kingdom in 2011, and it showed a high level of resistance to cephalosporins, carbapenems, aminoglycosides, and quinolones, while being susceptible to colistin and tigecycline (Hornsey et al., 2011). Following this discovery, two carbapenemase-producing *Enterobacteriaceae* (NDM-5 producing *E. coli* and NDM-1 producing *Klebsiella pneumoniae*) showing distinct hydrolytic activity against imipenem were isolated from a traveler from Bangladesh in 2013 and Indonesia in 2014, respectively (Nakano et al., 2014). Subsequently, in South Korea, NDM-9 and NDM-5 producing *Klebsiella variicola* and *E. coli* strains were recovered from a river in 2017 (Di et al., 2017) and patients in 2018 (Jhang et al., 2018), respectively, suggesting that environmental and clinical NDM-producing bacteria are in circulation.

The *bla*_*NDM*_ genes have been predominantly found in opportunistic pathogenic bacteria displaying resistance to multiple antimicrobials, particularly, *Enterobacteriaceae*, such as *E. coli*, *Klebsiella* sp., and *Enterobacter* sp. (Bush, 2010). Since the isolation of clinical NDM-1 producing *Acinetobacter* spp. and *Pseudomonas* spp. in 2012 (Bharadwaj, 2012), the occurrence of NDM-producing bacteria has been on the rise in various aquatic environments including river stream, wastewater treatment plants (WWTPs), and tap water (Walsh et al., 2011; Luo et al., 2013; Di et al., 2017). WWTPs have been suggested as potential hot spots for antibiotic resistance (Karkman et al., 2018). Contamination determinants from households, hospitals, farms, and other non-point source pollutions may play a role in selective pressure for the increase in antibiotic resistance, escalating antibiotic resistance that enables the development of multi-drug resistant (MDR), extensively drug resistant (XDR), and/or pan-drug resistant (PDR) bacteria, which make it increasingly difficult to treat infections.

In this study, we report the emergence of pathogenic, and highly carbapenem-resistant and XDR *E. coli* strain N7, isolated from the urban influent of Jungnang WWTP on the Han River located in Seoul, the capital city of South Korea. Whole-genome sequencing analyses of *E. coli* strain N7 indicated that 23 antibiotic resistance genes (ARGs) including *bla*_*NDM–*5_, a variant of NDM, were present in chromosome, plasmids, and contigs. Among them, seventeen were carried on two plasmids, which were formulated structurally in a manner of well-known conserved clusters with either class 1 integron and/or insertional sequences (ISs), suggesting that *E. coli* strain N7 can act as a carrier of ARGs in the aquatic environment.

## Materials and Methods

### Isolation and Identification of Carbapenem-Resistant Bacteria From a WWTP

The influent sample was collected from the Jungnang (JN) WWTP on the Han River, Seoul, South Korea in May of 2018 by using sterile bottles. After collection, the samples were immediately shipped to the laboratory under cool conditions (4°C) and filtered through a 0.22 μm pore size membrane filter (Advantec, Tokyo, Japan). The membranes were suspended in 10 mL of Mueller-Hinton (MH) broth (MBCell, Seoul, South Korea), thoroughly vortexed, and then processed with a serial dilution up to 10^–3^ times (10^0^, 10^–1^, 10^–2^, and 10^–3^). A 100 μL of sample of the MH broth was spread on mSuperCARBA (CHROMagar, France) agar plates and the plates were incubated at 37°C for 48 h. After incubation, the colonies on the plates were streaked on new MH agar plates containing 8 mg/L of meropenem to obtain a single colony of presumptive carbapenemase-producing bacteria. The isolate grown on the plates were taxonomically identified using 16S rDNA gene sequencing (Macrogen, Seoul, South Korea).

### Phenotypic and Genotypic Resistance Test

Eleven carbapenem resistance genes (*bla*_*IMP*_, *bla*_*VIM*_, *bla*_*NDM*_, *bla*_*SPM*_, *bla*_*AIM*_, *bla*_*DIM*_, *bla*_*GIM*_, *bla*_*SIM*_, *bla*_*KPC*_, *bla*_*BIC*_, and *bla*_*OXA–*48_) (Poirel et al., 2011) were screened using PCR detection from the presumptive carbapenemase-producing bacteria. The amplicons were sequenced (Macrogen) and identified using NCBI BLAST^1^. For the screened carbapenemase-producing bacteria, MDR to 16 antibiotics was determined using Kirby-Bauer disk diffusion, and resistance to colistin was determined using broth dilution methods. For MDR, the following antibiotic disks were used: ampicillin-sulbactam (10/10 μg), cefotaxime (30 μg), ceftazidime (30 μg), chloramphenicol (30 μg) ciprofloxacin (5 μg), colistin (2 mg/L), doripenem (10 μg), fosfomycin (200 μg), gentamicin (10 μg), levofloxacin (5 μg), meropenem (10 μg), netilmicin (10 μg), piperacillin (100 μg), tetracycline (30 μg), tobramycin (10 μg), and trimethoprim-sulfamethoxazole (1.25/23.75 μg) (Liofilchem, Roseto degli Abruzzi, Italy). Resistance to the antibiotics was determined according to the Clinical and Laboratory Standards Institute (CLSI) guideline (Clinical Laboratory Standars and Institue, 2016). Subsequently, MICs of 16 antibiotics for *E. coli* strain N7 were evaluated using the broth dilution method (Hasselmann, 2003).

### Whole Genome Sequencing

The genome was constructed *de novo* using PacBio sequencing data (Pacific Biosciences, Menlo Park, CA, United States). Sequencing analysis was performed at Chunlab Inc. (Seoul, South Korea). PacBio sequencing data were assembled with PacBio SMRT Analysis 2.3.0 using the HGAP2 protocol (Pacific Biosciences). The resulting contigs from PacBio sequencing data were circularized using Circulator 1.4.0 (Sanger Institute, Hinxton, Cambridgeshire, United Kingdom) (Yoon et al., 2017). Circular maps for plasmid structures and linear maps generated by Circulator 1.4.0 and geneCo (Jung et al., 2019), respectively, were manually modified. The chromosomal and plasmid origins of replication were identified using DoriC 5.0 and the plasmid types were determined by PlasmidFinder 1.3, using FASTA file (Carattoli et al., 2014). ARGs were identified using ResFinder (Zankari et al., 2012). Multi-locus sequence type (MLST) was determined by sequences of seven housekeeping genes (*adk*, *fumC*, *gyrB*, *icd*, *mdh*, *purA*, and *recA*) according to a previous description (Clermont et al., 2000). The WGS data were deposited in GenBank under the accession JABWPS000000000.

### Serotyping and Virulence Determinants

Carbapenemase-producing *E. coli* strain was serotyped with four O-antisera (O26, O111, O146, and O157) (SSI Diagnostica, Hillerød, Denmark) by incubation in MH broth for 16 h, boiling at 95°C for 15 min. Equal volume of the lysate and antisera were mixed in a 96-well culture plate, and then incubated at 52°C overnight. The agglutination of O-antigen and O-antisera was visually checked according to a previously described protocol (SSI Diagnostica). Virulence genes and serotype were determined from WGS data using VirulenceFinder and SerotypeFinder 2.0 (Carattoli et al., 2014; Joensen et al., 2015).

## Results

### Isolation and Identification of Carbapenem-Resistant Bacteria

Among the 50 isolates from the influent of JN WWTP, 24 isolates were presumptive carbapenem-resistant bacteria. The PCR detection of 11 carbapenemase genes (*bla*_*IMP*_, *bla*_*VIM*_, *bla*_*NDM*_, *bla*_*SPM*_, *bla*_*AIM*_, *bla*_*DIM*_, *bla*_*GIM*_, *bla*_*SIM*_, *bla*_*KPC*_, *bla*_*BIC*_, and *bla*_*OXA–*48_) revealed that only one isolate was positive for the *bla*_*NDM*_. This isolate, N7, was taxonomically identified as *E. coli* by 16S rDNA gene sequencing. MLST revealed that *E*. *coli* strain N7 belonged to ST746.

### Phenotypic Antimicrobial Resistance

*Escherichia coli* strain N7 showed resistance to ampicillin (MIC, 1,024 mg/L), cefotaxime (MIC, 256 mg/L), ceftazidime (MIC, 512 mg/L), ciprofloxacin (MIC, 1,024 mg/L), colistin (MIC, 8 mg/L), doripenem (MIC, 512 mg/L), fosfomycin (MIC, 1,024 mg/L), gentamycin (MIC, 512 mg/L), imipenem (MIC, 256 mg/L) levofloxacin (MIC, 256 mg/L), meropenem (MIC, 256 mg/L), netilmicin (MIC, 256 mg/L), piperacillin (MIC, 1,024 mg/L), tetracycline (MIC, 512 mg/L), tobramycin (MIC, 256 mg/L), and trimethoprim-sulfamethoxazole (MIC, 4/76 mg/L), but was susceptible to chloramphenicol (Table 1). Compared to the CLSI clinical breakpoint, *E. coli* strain N7 exhibited high level of resistance to eight classes of the antibiotics tested, except for trimethoprim-sulfamethoxazole. Regarding the extent of the antibiotic resistance up to 15 of 16 antibiotics tested, strain N7 is likely to be an XDR bacterium.

**TABLE 1:** MICs of antimicrobials tested for *E. coli* strain N7 compared with other *E. coli* strains.

No.	Antibiotics	MICs (mg/L) of *E. coli* strains				CLSI clinical breakpoint (mg/L)
		
		N7	QD28 (Rahman et al., 2014)	QD29 (Rahman et al., 2014)	EC405 (Zhu et al., 2016)	
1	GEN	512	32	≥256	–	16
2	CIP	1024	6	≥32	–	4
3	MEM	256	≥32	≥32	≥32	4
4	SXT	>4/76	–	–	–	4/76
5	CTX	256	≥256	≥256	≥256	4
6	CAZ	512	≥256	≥256	≥256	16
7	AMP	1024	–	–	–	32
8	PIP	1024	–	–	–	128
9	TET	512				16
10	FOF	1024	2	≥1024	–	256
11	NET	256	–	–	–	32
12	DOR	512	–	–	–	4
13	LVX	256	–	–	–	8
14	TOB	256	10	≥256	–	16
15	CHL	S	–	–	–	32
16	CST	8	0.38	0.5	–	2
17	IMP	256	–	–	–	4

*GEN: gentamicin; CIP: ciprofloxacin, MEM, meropenem; SXT, trimethoprim-sulfamethoxazole; CTX, cefotaxime; CAZ, ceftazidime; AMP, ampicillin; PIP, piperacillin; TET, tetracycline; FOF, fosfomycin; NET, netilmicin; DOR, doripenem; LVX, levofloxacin; TOB, tobramycin; CHL, chloramphenicol; CST, colistin; IMP, imipenem S, susceptible.*

### Determinants for Antimicrobial Resistance and Pathogenicity

WGS data showed that 23 ARGs were present in *E. coli* strain N7 and 21 ARGs were found on two incompatible plasmids and contigs, except for the *bla*_*CTX–*64_ and *mdf(A)* genes, which were located on the chromosome (Table 2). We found two plasmids in *E. coli* strain N7, identified as an IncX3 plasmid (pKJNI-5), and a hybrid plasmid consisting of IncHI2 and N-type (pKJNI-2).

**TABLE 2:** Genome features of *E. coli* N7 and its antimicrobial resistance genes.

Sequence type	Replicon	Origin of replication/plasmid incompatibility	Length (bp)	GC (%)	Resistance genes(*n* = 23)	Class of antimicrobials
ST746	Chromosome	*oriC*	4,614,699	50.84	*bla*_*CTX–*64_	β-Lactam
					*mdf(A)*	Macrolide
	Plasmid pKJNI-2	IncHI2, IncN	255,628	46.75	*bla*_*CTX–*14_	β-Lactam
					*aac(3)-IV, aadA1, aadA2, aph(3′)-Ia, aph(4)-Ia*	Aminoglycoside
					*sul1, sul2, sul3*	Sulfonamide
					*dfrA12*	Trimethoprim
					*fosA3*	Fosfomycin
					*oqxA, oqxB*	Quinolone
					*mph(A)*	Macrolide
					*floR, cmlA1*	Phenicol
	Plasmid pKJNI-5	IncX3	71,870	47.54	*bla*_*NDM–*5_	β-Lactam
	Contig 3		19,713	52.99	*tet(A)*	Tetracycline
	Contig 5		8,333	57.82	*erm(B)*	Macrolide
					*tet(M)*	Tetracycline
					*aadA22*	Aminoglycoside

**FIGURE 1:**
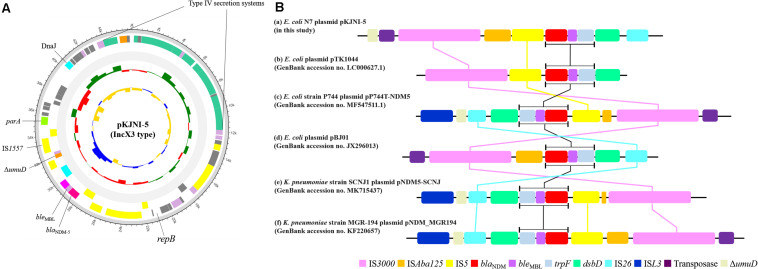
Structure of pKJNI-5 plasmid (IncX3 type) of *E. coli* N7 harboring *bla*_*NDM–*5_ **(A)** and comparative sequence analysis of regions of *bla*_*NDM–*5_ of *E. coli* N7 with other previously reported genetic structures of *bla*_*NDM*_ **(B)**. **(A)** From outermost to innermost ring of plasmid, forward and reverse CDS, track for rRNA and tRNA, GC Skew and GC Ratio was drawn. **(B)** The genetic environment of *bla*_*NDM–*5_ of (a) pKJNI-5 isolated in this study, (b) pBJ01, (c) pTK1044, (d) pNDM-MGR194, (e) pP744-T-NDM-5, (f) pNDM5-SCNJ were compared. Indication of each color was described below the genetic structures.

Figure 1A shows the structure of the IncX3 type plasmid pKJNI-5 containing the *bla*_*NDM–*5_ gene. As shown in the figure, the *bla*_*NDM–*5_ gene was always followed by a gene cluster composed of bleomycin resistance gene (*ble*_*MBL*_), phosphoribosyl anthranilate isomerase (*trpF*), and protein-disulfide reductase (*dsbD*), as previously reported (Nakano et al., 2014; Zhu et al., 2016; Ho et al., 2018; Yuan et al., 2019). The gene cluster of *bla*_*NDM–*5_-*ble*_*MBL*_-*trpF*-*dsbD* was also bracketed by IS*3000-*IS*Aba125-*IS*5* in the upstream region and IS*26* in the downstream region (Figure 1B), which was also well conserved among diverse bacteria with the *bla*NDM-5 and *bla*NDM-1 genes (Nakano et al., 2014; Zhu et al., 2016; Ho et al., 2018; Yuan et al., 2019). It should be noted that IS*3000* was always found upstream of the gene cluster of *bla*_*NDM–*5_-*ble*_*MBL*_-*trpF*-*dsbC/D* regions among diverse bacteria. In addition, except in *E. coil* pTK1044, IS*26* is always located downstream of the gene cluster. Figure 1A shows the presence of a type IV secretion system (*virD2-virB1-virB4-virB5-virB6-virB8-virB9-virB10-virB11-virD4*) at a site opposite that of *bla*_*NDM–*5_ on the plasmid pKJNI-5.

**FIGURE 2:**
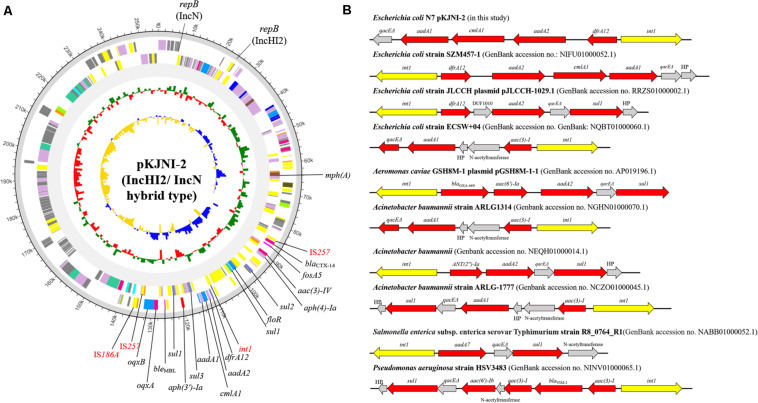
Circular map and comparative sequence analysis of pKJNI-2 plasmid (IncHI2/IncN hybrid type) and comparison of class 1 integron structures. **(A)** From outermost to innermost ring of plasmid, forward and reverse CDS, track for rRNA and tRNA, GC Skew and GC Ratio was drawn. ARGs and mobile genetic elements were indicated in black and red colors, respectively. **(B)** Class 1 integron structure of pKJNI-2 was compared with those previously reported. The *int1* gene and ARGs were colored in yellow and red colors, respectively.

Figure 2A shows the IncHI2/N hybrid-type plasmid pKJNI-2, which carries 16 ARGs {aminoglycosides [*aac(3)-IV, aadA1, aadA2, aph(3′)-Ia*, and *aph(4)-Ia*], β-lactams (*bla*_*CTX–*14_), fosfomycin (*fosA3*), macrolide [*mph(A)*], phenicols (*floR* and *cmlA1*), quinolones (*oqxA* and *oqxB*), sulfonamide (*sul1, sul2*, and *sul3*), and trimethoprim (*dfrA12*)}. Even in the presence of the phenicol resistance gene on the IncHI2/N hybrid plasmid, *E. coli* strain N7 was susceptible to chloramphenicol. IS*257* brackets 15 ARGs except for macrolide [*mph(A)*], and contains a class 1 integron, which carries resistance genes to aminoglycoside (*aadA1* and *aadA2*), chloramphenicol (*cmlA1*), and trimethoprim (*dfrA12*) (Figure 2B). The gene cassette associated with class 1 integron of *E. coli* strain N7 was compared with that of previously submitted genomic data of other bacterial strains including *Aeromonas caviae*, *A. baumannii*, *Salmonella* Typhimurium, *P. aeruginosa* (Figure 2B). It shows similar patterns of carrying a narrow range of resistance genes to aminoglycoside, β-lactam, chloramphenicol, sulfonamide, and trimethoprim.

In addition, *E. coli* strain N7 carries the following eight virulence factors: adhesion-associated molecules (*fimH*, *flu*, *ecpABCDE*, and *sfmA*), and toxins-encoding genes (*hlyE* and *gadA)*. *E*. *coli* strain N7 belongs to H37 but O-serotype was not determined.

## Discussion

In the present study, we report on the emergence of XDR *E. coli* strain N7 which is positive for *bla*_*NDM–*5_ and characterization of the genetic context of ARGs, including *bla*_*NDM–*5_. Since the discovery of NDM in a Swedish patient who traveled to India, its variants have grown to 28 different types from diverse bacteria, mostly isolated from clinical samples. In South Korea, NDM-5 producing *Enterobacteriaceae* have been reported only in clinical environments (Park et al., 2016, 2019; Kim et al., 2020), and NDM-9 producing *K. variicola* were found in river (Di et al., 2017).

*Escherichia coli* strain N7 belonging to ST746 isolated from the urban influent of JN WWTP shows a variant of the NDM, NDM-5 type. From the WGS, we identified two plasmids such as a narrow host range plasmid IncX3 (Johnson et al., 2012) and a hybrid IncHI2/N. The narrow host range plasmid IncX3 carries a cluster structure of 5′-IS*3000*-DIS*Aba125*-IS*5*-*bla*_*NDM–*5_-*ble*_*MBL*_-*trpF*-*dsbD*-IS*26*-3′ containing the *bla*_*NDM–*5_ gene. Figure 1A shows the composition of ISs, which cassettes structural genes of 5′-*bla*_*NDM–*5_-*ble*_*MBL*_-*trpF*-*dsbD*-3′ (Liu et al., 2013; Nakano et al., 2014; Zhu et al., 2016; Yuan et al., 2019) with a minor change in the presence and absence of IS*5* and the extent of truncated IS*Aba125* among the analyzed *E. coli* and *K. pneumoniae* strains. The question is still remained why the structural genes of 5′-*ble*_*MBL*_-*trpF*-*dsbD*-3′ with *bla*_*NDM–*5_ are always clustered together. In addition, the IncX3 type plasmid in *E. coli* strain N7 also contains a type IV secretion system (*virD2-virB1-virB4-virB5-virB6-virB8-virB9-virB10-virB11-virD4*) located at a site opposite that of *bla*_*NDM–*5_. It should be noted that the type IV secretion system has also been hypothesized to be involved in horizontal gene transfer between other bacteria (Juhas et al., 2008). Taken together, *E. coli* strain N7 is likely to have a system to transfer recently emerged *bla*_*NDM–*5_ gene to other bacteria due to multiple ISs and type IV secretion system, although it contains the narrow host range vector system (Liakopoul et al., 2018).

It is known that *E. coli* ST746 carries extended-spectrum β-lactamase (ESBL) genes from fishes (Sellera et al., 2018) and human patients (Wu et al., 2018). In this study, *E. coli* strain N7 harbored ESBL and eight virulence factors. Surprisingly, *E. coli* strain N7, which showed MIC of meropenem at 256 mg/L, was also resistant to several antibiotics with very high MIC values for the tested antimicrobials (Table 1), compared to other NDM-5 producing *E. coli* strains (Hornsey et al., 2011; Rahman et al., 2014; Zhu et al., 2016; Jhang et al., 2018). This XDR pattern can be explained by the presence of several resistance genes located on the broad host range plasmid (Figure 2; Zhao et al., 2018). Therefore, the presence of XDR *E. coli*, isolated from the influent of WWTP located in a city, along with the carbapenem-resistance gene raises public health concerns due to the possible dissemination of ARGs to other pathogenic bacteria, and difficulty in treatment of infections. Indeed, XDR pathogenic *E. coli* strains have been reported from human patients (harboring *bla*_*KPC–*2_) (Jeong et al., 2018) and from chickens (co-producing *bla*_*NDM*_ and *mcr-1*) (Lv et al., 2018), increasing the likelihood of infectious disease outbreaks. The characteristics of XDR *E. coli* strain N7 can be attributed to the presence of corresponding resistance genes located on two plasmids of an IncX3 and a hybrid IncHI2/N. The occurrence of the IncHI2 plasmid has been frequently reported in *Salmonella* strains with multiple ARGs (Chen et al., 2016). In our experiments, most of the resistance genes were found on the hybrid plasmid IncHI2/N of *E. coli* strain N7, containing diverse resistance determinants, including aminoglycoside [*aac(3)-IV, aadA1, aadA2, aph(3′)-Ia*, and *aph(4)-Ia*], β-lactam (*bla*_*CTX–*64_, *bla*_*CTX–*14_, and *bla*_*NDM–*5_), fosfomycin (*fosA5*), macrolide [*mdf(A)* and *mph(A)*], phenicol (*floR* and *cmlA1*), quinolone (*oqxA* and *oqxB*), sulfonamide (*sul1, sul2*, and *sul3)*, and trimethoprim (*dfrA12*).

## Conclusion

In conclusion, NDM-5 producing *E. coli* strain N7, which shows a high level of carbapenem resistance and an XDR pattern, was found in the megacity influent of Jungnang WWTP, Seoul, South Korea. Our findings suggest that pathogenic XDR *E. coli* originating from urban activities may be disseminated into the river from WWTP and is a potential carrier or spreader of ARGs, including emerging carbapenemase genes. Thus, we need to focus on the continuous surveillance of carbapenemase-producing bacteria in diverse environments.

## Data Availability Statement

The datasets presented in this study can be found in online repositories. The names of the repository/repositories and accession number(s) can be found in the article/supplementary material.

## Author Contributions

HS: experiment, data analysis, and manuscript writing. YK: methodology. DH: revision of manuscript. H-GH: overall revision, methodology, and data analysis. All authors contributed to the article and approved the submitted version.

## Conflict of Interest

The authors declare that the research was conducted in the absence of any commercial or financial relationships that could be construed as a potential conflict of interest.

## Footnotes

1 http://www.ncbi.nlm.nih.gov
